# Quercetin-Embedded Gelastin Injectable Hydrogel as Provisional Biotemplate for Future Cutaneous Application: Optimization and In Vitro Evaluation

**DOI:** 10.3390/gels8100623

**Published:** 2022-09-29

**Authors:** Mazlan Zawani, Manira Maarof, Yasuhiko Tabata, Antonella Motta, Mh Busra Fauzi

**Affiliations:** 1 Centre for Tissue Engineering & Regenerative Medicine, Faculty of Medicine, Universiti Kebangsaan Malaysia, Kuala Lumpur 56000, Malaysia; 2 Laboratory of Biomaterials, Department of Regeneration Science and Engineering, Institute for Life and Medical Science (LiMe), Kyoto University, 53 Kawara-cho Shogoin, Sakyo-Ku, Kyoto 606-8500, Japan; 3 Department of Industrial Engineering, University of Trento, via Sommarive 9, 38123 Trento, Italy

**Keywords:** chronic skin wound, quercetin, injectable, hydrogel, gelatin, elastin, regenerative medicine, tissue engineering, biomaterial, in vitro

## Abstract

Chronic wounds have become an epidemic in millions of patients and result in amputations. In order to overcome this, immediate treatment is a realistic strategy to minimize the risk of complications and aid in the healing rate of the cutaneous wound. Functionalized engineered biomaterials are proven to be a potential approach to embarking on skin wound management. Thus, this study aimed to evaluate the efficacy of a quercetin-embedded gelatin–elastin (Gelastin) injectable hydrogel to act as a provisional biotemplate with excellent physicochemical properties, to be utilized for future cutaneous application. Briefly, the hydrogel was homogenously pre-mixed with genipin (GNP), followed by the incorporation of quercetin (QC). The physicochemical properties comprised the contact angle, swelling ratio, crosslinking degree, enzymatic biodegradation, and water vapor transmission rate (WVTR), as well as chemical characterization. Energy-dispersive X-ray (EDX), XRD, and Fourier transform infra-red (FTIR) analyses were conducted. Briefly, the findings demonstrated that the crosslinked hybrid biomatrix demonstrated better resilience at >100%, a contact angle of >20°, a swelling ratio average of 500 ± 10%, a degradation rate of <0.05 mg/hour, and a successful crosslinking degree (<70%free amine group), compared to the non-crosslinked hybrid biomatrix. In addition, the WVTR was >1500 g/m^2^ h, an optimal moisture content designed to attain regular cell function and proliferation. The outcomes convey that Gelastin-QC hydrogels deliver the optimum features to be used as a provisional biotemplate for skin tissue engineering purposes.

## 1. Introduction

The estimated Medicare costs for acute and chronic wound treatments have fluctuated between USD 28.1 billion and USD 96.8 billion. Exorbitant expenditure was sourced to surgical wounds and diabetic foot ulcers (DFU), with an elevated trend toward outpatient wound care-associated costs compared to inpatient. The inflated costs of healthcare, the geriatric population, difficult-to-treat infection threats such as biofilms, and the global threat of diabetes and obesity have substantiated chronic wounds as an economic, clinical, and social challenge [1]. Chronic wounds exhibit biofilm growth, protease elevation, and bacterial clusters as they are usually stalled in the inflammatory phase. The inhibitors are dominated by protease, which leads to the destruction of ECM, hence elevating the protease’s proliferation and accelerating the inflammation phase. The progression of the following event triggers ROSs to inflate, resulting in the malfunctioning of ECM proteins and premature cells [2]. Damaged tissues sustain a complex healing process in order to self-repair prior to pathological injuries. Throughout the process, the body’s immune response engenders several growth factors, cells, free radicals, and pro-oxidant species. In low concentrations, the pro-oxidant species assist in variegated physiological facets [3]; however, circumspectly over spatiotemporal factors, the inundation of pro-oxidant species as well as free radicals on the wound site may be lethal. Therefore, several studies have propounded the integration of antioxidant and/or free radical scavenging substances into biomaterials to encourage as well as assist body function repair and tissue regeneration [4].

With technological advancement, a number of state-of-the-art therapies are implemented for these types of wounds, for instance, the development of negative-pressure wound therapy, skin substitutes, hyperbaric oxygen, the fabrication of novel growth factors embedded in wound dressings, and tissue-derived engineered biomaterials. Functionalized biomaterials, play an important role in tissue engineering for tissue repair and regeneration. These engineered scaffolds are an excellent temporary ECM with biological mimicry and are highly biocompatible, whereas engineered hydrogels have the ability to alter their properties and are engineered to resemble the soft tissues of ECM [5]. Hydrogels hold unique biomimicry features of flexibility and the ability to create a three-dimensional network via crosslinking based on the organs’ mechanical stimuli; they aim to assist with cell adhesion, proliferation, and migration, as well as cell and molecule delivery, making them appealing for tissue engineering implementation [6]. Natural biomaterials such as fibrin, collagen, chitosan, cellulose, gelatin, and alginate are favored in developing hydrogels as they are substantially abundant in ECM arrangements [7,8,9,10,11]. Among the natural biomaterials, gelatin has been considered a popular polymeric biomaterial for multipurpose scientific applications, mainly in wound healing and drug delivery [12]. Gelatin, a substance derived from type 1 collagen and an abundance of amino acids able to sustain and accelerate cell proliferation, has been broadly used to mimic the ECM structural protein. It is commonly utilized in this field due to its high abundance, low cost, good cellular compatibility, biodegradability, enhanced tissue regeneration, good absorption, and low antigenicity [13]. Functionalized hydrogels have been widely proven to embark on a potential strategy of cutaneous wound management, and active constituents’ embedded biomaterials play a key role in the advanced era of tissue engineering [14]. Thus, it is hoped that incorporating quercetin to combat delayed wound healing in these chronic conditions will succeed as it is widely proven to exhibit angiogenesis, epithelial cell proliferation, and accelerated wound closure [15,16]. Moreover, the addition of elastin a fatigue-resistant protein in the hybrid hydrogel, was intended to aid with elasticity, and is proven to exert multiple advantages in wound healing by improving the skin’s mechanical elasticity; the reduction in contraction and scar formation participates in cell signaling and regulates vascular morphogenesis deformation [17,18]. Due to the low mechanical properties of gelatin, genipin, an agent extract from the gardenia fruit with rich blue pigment formation upon reaction with the amino group, is introduced as the crosslinker. It is desirable for its low toxicity and spontaneous crosslinking ability in the presence of oxygen [19].

The work aimed to evaluate the efficacy of the fabricated Gelastin quercetin-embedded hydrogel to deliver the optimum outputs, to be utilized as a provisional biotemplate of skin tissue for wound healing purposes. Briefly, the injectable hydrogel is a combination of 7% gelatin and 2% elastin, with the addition of QC as well as GNP (0.1%) as the crosslinker. Our proposed idea was to create a one-time-application injectable hydrogel; hence, several tests were conducted to characterize the ideal hydrogel, whereby Energy-Dispersive X-ray (EDX), X-ray Diffraction (XRD), and Fourier transform infrared (FTIR) methods were used for chemical characterization. The physicochemical properties, contact angle, swelling ratio, crosslinking degree, enzymatic biodegradation, and water vapor transmission rates were determined.

## 2. Results and Discussion

### 2.1. Optimization and Physical Characteristics

**Figure 1:**
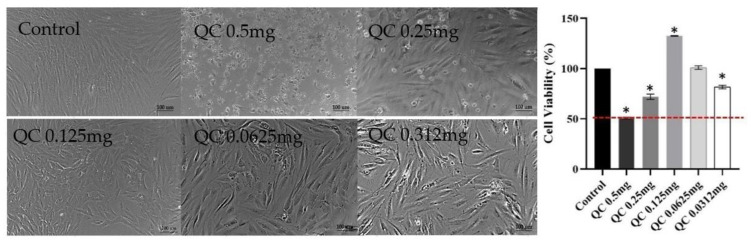
The MTT Cell Toxicity assay. The effect of quercetin at diverse concentrations (0.312, 0.0625, 0.125, 0.25, and 0.5 mg/mL) on the morphology of human dermal fibroblasts (HDFs). Quantitative analysis of the percentage of HDFs treated with serial concentrations of quercetin (0.312, 0.0625, 0.125, 0.25, and 0.5 mg/mL) at 24 h. No treatment group acted as the control group. * *p* < 0.05 indication of quercetin-treated vs. no-treatment.

**Figure 2:**
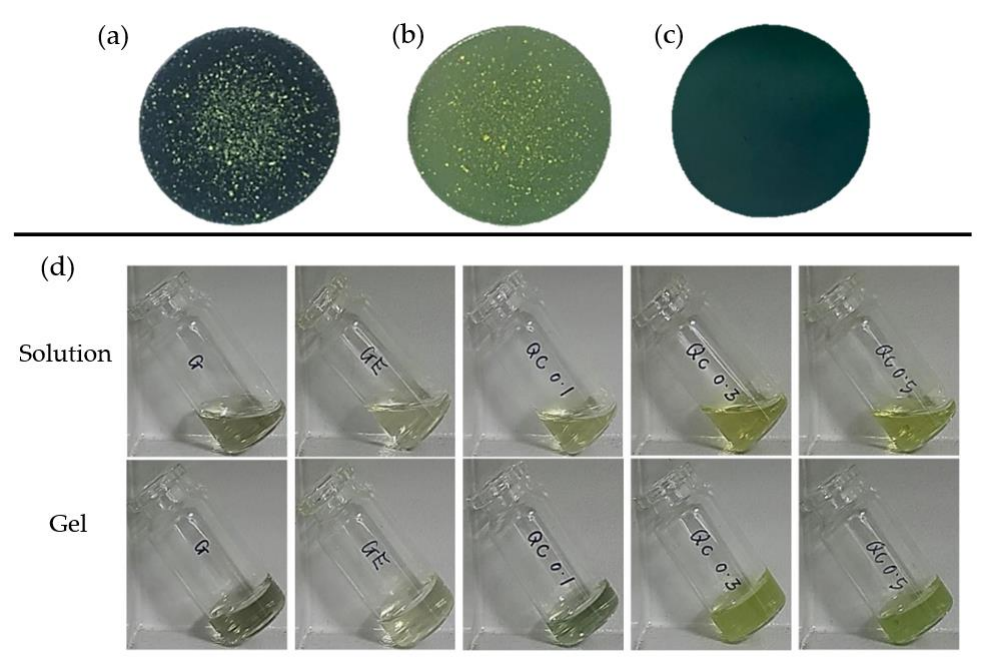
Optical images of quercetin Gelastin hydrogel with different types of solvent: Milli-Q water (**a**), 90% ethanol (EtOH) (**b**), and dimethyl sulfoxide (DMSO) (**c**). (**d**) Tilt test. The sol–gel transition of hybrid hydrogels at room temperature.

Injectable hydrogels, which induced a crosslinking response prior to implantation onto the defect area, have the ability to be transported into direct contact with the surrounding structure regardless of the complexity of the shape. Various ranges of gelatin concentrations (5% *w*/*v* and 7% *w*/*v*) with different swelling points were utilized and the fabricated hydrogels were compared to optimize the aim of achieving 3 min of polymerization prior to implantation. A comparison was made between homogeneity, odor, gelation time, and appearance (Table 1). The MTT cell toxicity assay is vital in developing tissue-engineered biomaterial, as the maximal concentration of quercetin identified as cytotoxic can be discerned. Furthermore, according to the ISO standard, the concentration which sustains >50% cell viability is considered non-cytotoxic. The MTT assay specified that all the selected quercetin concentrations sustained high cell viability above 50%. At the same time, the optimum concentration was 0.125 mg/mL of quercetin (Figure 1).In general, QC is solubilize in DMSO as it gives the most stable form when incorporated into the hydrogels as can be seen in Figure 2a–c. Whereas, hydrogels with 7% (*w*/*v*) gelatin concentration achieved a polymerization and gelation time point of within 3 min upon implantation, when the gelatin is soaked in dH_2_O for 15 min, 30 min, and 1 h, respectively, whereas the whole 5% (*w*/*v*) gelatin concentration group did not achieve gelation time within the target time point (Figure 2d). Hence, a 10 min swelling duration was chosen for the study as it had the best gelatin solution consistency during the optimization analysis.

### 2.2. Chemical Characterization

#### 2.2.1. Fourier Transform Infra-Red (FTIR)

**Figure 3:**
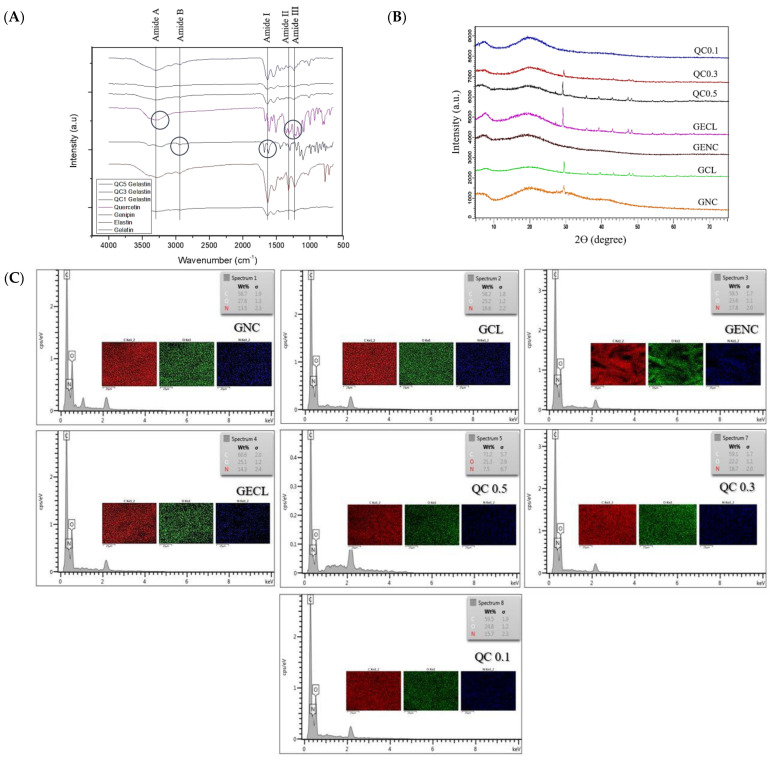
(**A**) FTIR intensity and (**B**) XRD analysis of the hybrid hydrogels. (**C**) EDX Mapping.

FTIR produces a distinctive molecular fingerprint for the identification of polymers and crosslinkers and their chemical bonds. Fundamentally, Amides A and B and Amides I-VII are the most prominent peaks in protein [20]. The IR spectra obtained from the analysis demonstrate the vibrational band of gelatin hydrogels positioned within the amide region (Figure 3A). The 3232–3337 cm^−1^ peaks correspond to OH and Amide A, representing the pairing of NH stretching and H-bonds. Meanwhile, 1620–1640 cm^−1^ characterizes Amide I, the most prominent band in the identification of proteins’ secondary structures, which represent either C=O stretching or the pairing of H-bonds and COO [21]. Whereas, peaks at 1530 cm^−1^ and 1635 cm^−1^ correspond to Amides I and II of elastin,1300–1370 cm^−1^ band represents the C-OH bond, the analogous fingerprints of genipin and gelatin that identified in all the hydrogels. With the addition of quercetin, the absorption of the C-O band (1207 cm^−1^), the C-C group (1591 cm^−1^), the -OH band (3406 cm^−1^), and the C-H group (2800–2900 cm^−1^) was present; these are similar to the native quercetin vibrational band [22]. The crosslinked quercetin-embedded hydrogels have broadened peaks which, when repositioned to lower wavenumber, indicate functional group interaction between all the compositions (gelatin, quercetin, genipin, and elastin).

#### 2.2.2. X-ray Diffraction (XRD) 

The amorphous hydrogels and the crystallinity phase can be seen using XRD analysis. The crystallinity of hydrogel increases when genipin is added as genipin is highly crystalline compared to the non-crosslinked GNC and GENC (18.1% and 37.7% crystallinity, respectively). However, with the incorporation of QC, the crystallinity attains the highest percentage in QC 0.5 (46.7%), followed by QC 0.3 (42.0%) and QC 0.1 (40.3%). This phenomenon can be explained by the highly crystalline structure trait of pure quercetin [23]. The occurrence of the prominent peak at 2θ (2Theta) = 28° and a small sharp peak at 30° to 40° suggests the genipin structure’s sustainability, and that all the hybrid hydrogels exert amorphous structures above 50% (Figure 3B) (Table 2).

#### 2.2.3. Dispersive X-ray (EDX)

EDX identified the elemental composition of the materials, and the electron image exhibited three key elements: oxygen, carbon, and nitrogen. With the addition of quercetin, there is a slight increase in carbon in the QC-embedded hydrogels as it is the most elemental composition of QC (Figure 3C) (Table 3). The EDX mapping shows a homogenous compositional mixture of all the hydrogels with a higher value of carbon (QC 5: 62.9 ± 2.50, QC 3: 62.6 ± 4.81, and QC 1: 60.2 ± 2.12) and oxygen (QC 5: 18.3 ± 1.79, QC 3: 18.2 ± 2.56, QC 1: 18.0 ± 2.34), respectively, in the EDX result of QC-embedded hydrogel, similarly to what was reported for flavonoid incorporation. 

### 2.3. Morphological Study

**Figure 4:**
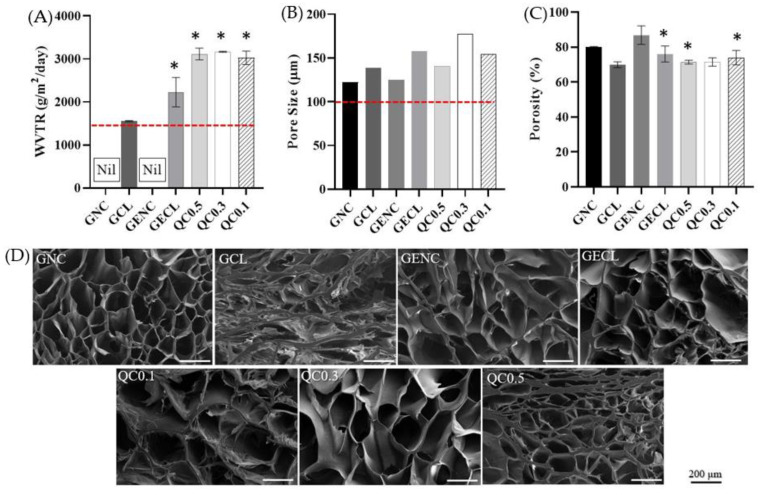
WVTR, pore size, porosity, and pore morphology. (**A**) WVTR of quercetin-embedded Gelastin hydrogels shows a good WVTR range between 2000 and 3000 g/m^2^/day. (**B**) Average pore sizes of hybrid hydrogels are within the range of 50–200 µm. (**C**) Porosity of non-crosslinked hydrogels is >60%, significantly higher than crosslinked hydrogels. (**D**) SEM images reveal interconnected porous structures for cross-sectional morphology of the hybrid gelatin hydrogels. * significant difference (*p* < 0.05).

Hydrogels are crosslinked macromolecular networks formed of hydrophilic polymers swollen in water or biological fluids. Upon implantation, hydrogel porosity allows for local angiogenesis to occur, which is a key requirement for vascularized tissues. From the scanning electron microscope, we are able to capture the cross-section view of the hydrogels interconnected pores. The GNC group has the smallest pore size average of 116 ± 55 μm compared to all the groups (Figure 4D). The gelatin and genipin concentrations play significant roles in regulating the pore dimensions. However, in no case does the incorporation of quercetin into the gelatin hydrogel significantly affect the morphology and pore size of the hydrogel (Figure 4D). In comparison to non-crosslinked gelatin hydrogel with an average pore size of 116 ± 55 μm, 125 ± 37 μm genipin crosslinked gelatin hydrogels show an increase in the average pore size of GCL (132 ± 43 μm); GECL (188 ± 90 μm); QC 0.1 (135 ± 24 μm); QC 0.3 (220 ± 52 μm); and QC 0.5 (142 ± 28 μm). An ideal pore size for adult mammalian skin regeneration ranges between 20 and 125 μm, and an increase in pore size has been proven to elevate cell ECM secretion and cell proliferation [24] (ANOVA and Tukey post hoc analysis, *p* < 0.05).

For porosity, due to the small, interconnected structure of the NC hydrogels, these groups obtain a higher porosity percentage for GNC (78 ± 28%) and GENC (85± 24%) compared to the crosslinked group. The GENC hydrogel attains the highest porosity, which shows that the addition of elastin has altered the hydrogel’s structure by adding elasticity.

#### Water Vapor Transmission Rate (WVTR)

Wound dressings are used to demonstrate suitable WVT properties for developing a favorable environment for rapid wound healing. Thus, WVTR is vital to evaluate hydrogel diffusion ability, as adapted from the literature, the average water vapor transmission rate of normal human skin is 204 ± 12 g/m^2^/day, whereas first-degree burn is 279 ± 26 g/m^2^/day, and skin injury is 5138 ± 202 g/m^2^/day, respectively [25,26]. The rate of WVT for biotemplates should not be too low or too high, as the accumulation and overflowing of exudate may occur when the transmission rate is low, whereas high permeability may eventually lead to extreme dehydration of the wound [27]. There are a few studies suggesting that the most optimal WVTR for a skin biotemplate is between 2000 and 2500 g/m2/day, to maintain good moisture retention without excessive dehydration of the wound [28]. Our hydrogels successfully retain the WVTR range from 2000 to 3000 g/m^2^/day (Figure 4A). Altogether, the measurements demonstrate quercetin-embedded hydrogels obtain WVT of 3017 ± 764 g/m^2^/day, 3157 ± 100 g/m^2^/day, and 2916 ± 703 g/m^2^/day suitable rate for wound-dressing applications.

### 2.4. Physical Properties

#### 2.4.1. Gross Appearance

The gross appearance of the hydrogels shows clear translucent hydrogels, which indicate NC Gelastin hydrogels (NC: non-crosslinked) whereas when crosslinked, the hydrogels emit a bluish-green appearance, showing a successful crosslinking effect of CL Gelastin hydrogels (CL: crosslinked) (Figure 4A). Genipin readily undergoes an impetuous reaction with primary amine in the presence of oxygen, hence producing water-soluble blue pigments, whereas with the addition of yellow quercetin pigments, the quercetin-embedded hydrogels appear to be green in color. 

#### 2.4.2. Degree of Crosslinking

**Figure 5:**
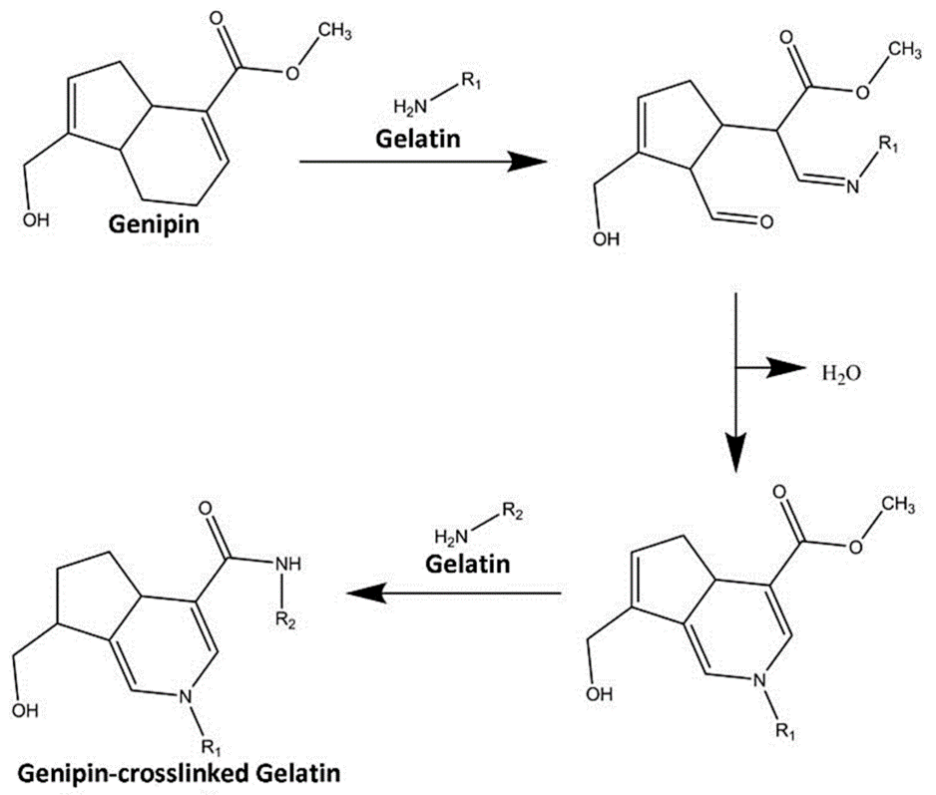
Crosslinking mechanism of genipin. Ring-opening reaction is initiated by amino group through nucleophilic attack towards the genipin olefinic carbon atom. The image, obtained from Nike et al. [30], was licensed under Creative Commons CC BY 4.0.

The analysis of the degree of crosslinking is expressed as the reduction in the free ε-amino group via colorimetry. The crosslinking mechanism between genipin and the amino group involves a nucleophilic attack of the gelatin amino group toward the genipin C-3 olefinic carbon atom, where the formation of heterocyclic amino linkage crosslinking occurs, initiated by the opening of the dihydropyran ring (Figure 5). From the graph provided in Figure 3, the data demonstrate that the addition of 0.1% GNP is sufficient to crosslink >50% ε-amino group as can be seen in the crosslinked hydrogels (G 51.36 ± 0.87%; GE 61.9 ± 0.11%). However, with the addition of QC the QC-embedded hydrogels acquire a higher percentage of crosslinking, where the highest concentration of embedded QC 0.5 has the highest crosslinking degree of 75.52 ± 0.43% in comparison with the whole group, followed by QC 3 (71.37 ± 0.54%) and QC 1 (61.64 ± 0.54%); this may be supported by the fact that quercetin has a small ratio of crosslinking ability, as reported by Greco et al. [29].

#### 2.4.3. Contact Angle 

**Figure 6:**
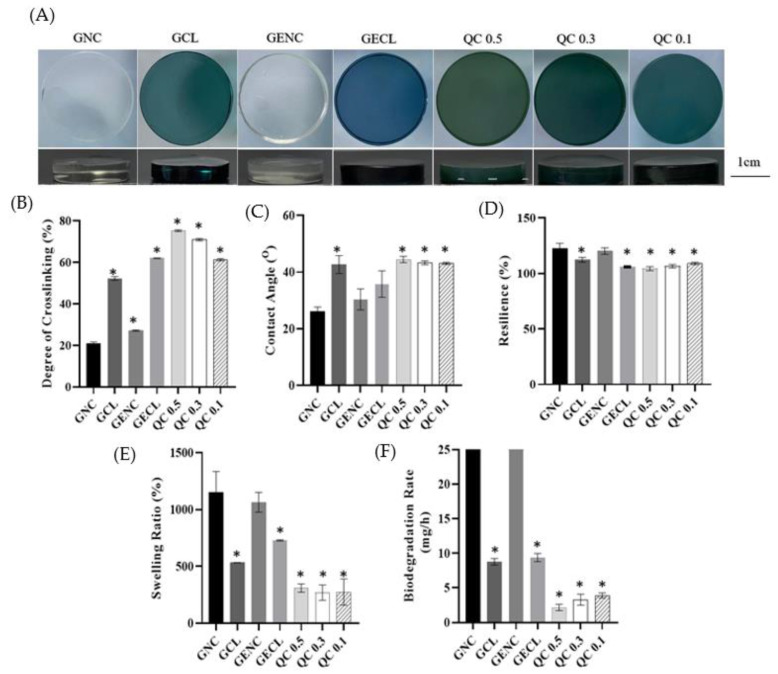
(**A**) The gross appearance of the hydrogels after polymerization and the physical characterization of the hybrid hydrogels, (**B**) crosslinking degree, (**C**) contact angle analysis, (**D**) resilience, (**E**) swelling ratio, (**F**) in vitro biodegradation. * significant difference (*p* < 0.05).

This analysis is important for assessing the wettability and adhesiveness of the biomaterial. In general, contact angles above 90° correspond to a hydrophobic surface, whereas angles below 90° represent a hydrophilic surface. From the data analysis, all the hydrogels obtain a contact angle of less than 90°; this shows hydrophilicity of the hydrogels, which are important in aiding in cell attachment for future application (Figure 6C). The non-crosslinked hydrogels, GNC (27.10 ± 1.53°) and GENC (28.68 ± 3.71°), have a lower contact angle in comparison to the crosslinked hydrogels, GCL (40.31 ± 3.15°) and GECL (40.60 ± 4.68°), whereas the quercetin-embedded hydrogels, QC 5 (43.23 ± 1.10°), QC 3 (43.54 ± 0.60°), and QC 1 (43.01 ± 0.36°), obtain higher contact angles compared to the other groups. The increased water contact angle suggests that the introduction of QC significantly reduces the surface hydrophilicity due to the change in surface composition; as, QC is also known as a hydrophobic drug [23].

#### 2.4.4. Resilience

This assay aimed to test the ability of the hydrogels to retain their original shape after applying pressure; the analysis propelled all the crosslinked hydrogels to retain their original shape after applying pressure, with approximately a 100% resilience percentage (Figure 6D) (Table 4), showing tremendous mechanical properties. If it exceeded 100%, the hydrogel had a higher possibility of bursting and disrupting the hydrogel matrix; hence, genipin, in conjunction with gelatin, succeeded in creating an elastic and resistant gel. Moreover, the high resilience might also be due to the well-defined matrix structure yield via crosslinking, where the NC (GNC 127 ± 4.7 %; GENC 124 ± 2.6 %) group acquired the highest percentage. 

#### 2.4.5. Swelling Ratio

The capacity of hydrogels to retain and adsorb water is one of the crucial parameters to be evaluated in wound healing, to assess the potential ability of the hydrogels to absorb excess wound exudates, and hence, maintain a suitable microenvironment in the wound. In the non-crosslinked groups, GNC (1125.67 ± 275%) and GENC (1244.86 ± 102%), the swelling rate was higher compared to the quercetin-embedded group, which attained QC 0.5 (350.85 ± 94%), QC 0.3 (344.73 ± 66%), and QC 0.1 (405.67 ± 30%), respectively (Figure 6E). This is due to the formation of a covalent bond via in situ crosslinking between genipin and gelatin constructs, creating a much more prominent microstructure, limiting the crosslinked hydrogels’ expansion ability.

#### 2.4.6. In Vitro Biodegradation 

Collagenase type I has been utilized for enzymatic biodegradation to mimic human body fluid. Our aim is to create a one-time-application biotemplate; hence, it is preferable for the hydrogels to be able to sustain themselves for at least 7 days before being fully degraded. From the analysis (Figure 6F), the NC hydrogels were fully degraded within an hour; however, we successfully obtained the desired degradation rate for all the CL hydrogels with GCL (8.36 ± 0.46 mg/h) and GECL (9.33 ± 0.59 mg/h), and with the addition of QC, the hydrogels showed slower degradation rates of QC5 (2.19 ± 0.47 mg/h), QC3 (2.37 ± 0.81 mg/h), and QC1 (3.06 ± 0.36 mg/h); this might be a small fraction of the crosslinking property exerted by QC [29]. 

#### 2.4.7. Rheological Characterization

**Figure 7:**
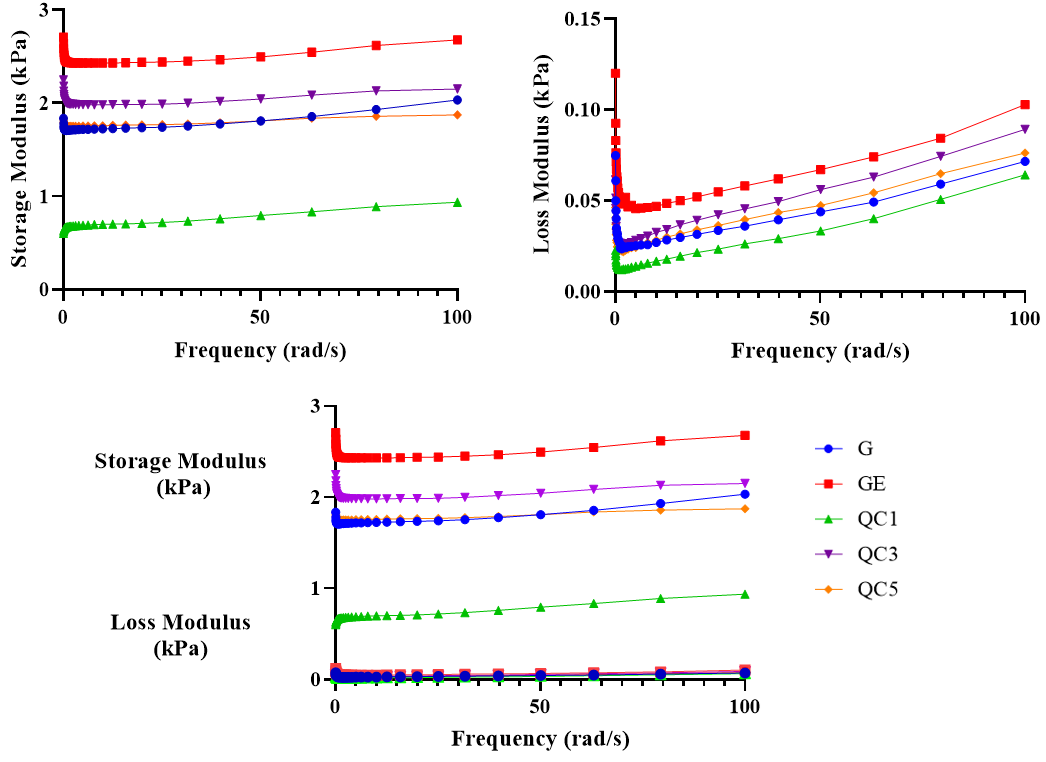
Rheological characterization of Gelastin hydrogels. Storage modulus G′ and loss modulus G′′ under different angular frequencies. The trend shows that the storage modulus and the loss modulus of the hydrogels increase with an increase in frequency.

The viscoelasticity of Gelastin hydrogels was assessed via rheology. All the hydrogels exhibit a higher storage modulus (G′) compared to the loss modulus (G″) at constant room temperature. There is no significant difference in the storage modulus between Gelastin and QC-embedded hydrogels. All of the hydrogels retain G (2 ± 0.07 kPa), GE (2.6 ± 0.08 kPa), QC1 (0.9 ± 0.073 kPa), QC3 (2.1 ± 0.069 kPa), and QC5 (1.8 ± 0.03 kPa), respectively, where 1 to 7 kPa is acceptable for skin and soft tissue substitutes. Increasing the amount of QC in the hydrogels does not affect the viscoelasticity of the hydrogels, as shown in Figure 7. The storage modulus represents energy stored in the hydrogel’s elastic structure, whereas the loss modulus represents the viscous part of the energy dissipated in the hydrogel; hence, a higher storage modulus compared to the loss modulus represents a highly elastic material [31]. Moreover, the increase in storage and loss moduli also indicates an increase in both elastic and viscous effects, which may later affect the droplet formation process during the injection. GE is the most viscous as it obtains the highest loss modulus (0.1 ± 0.018 kPa) at a 100 rad/s angular frequency; however, there are no significant differences obtained in the entire hydrogel group as all the other hydrogels acquire G (0.07 ± 0.01 kPa), QC 1 (0.06 ± 0.012 kPa), QC 3 (0.08 ± 0.015 kPa), and QC 5 (0.07 ± 0.013 kPa), respectively.

### 2.5. Cell Bioscaffold Interaction

#### LIVE/DEAD™ Cell Viability

The LIVE/DEAD™ Cell Viability Assay consists of calcein and EthD-1. Calcein easily penetrates and stains the cytoplasm of healthy live cells, whereas EthD-1 has a high affinity toward the cell’s DNA and emits red fluorescence staining. As shown in Figure 7, a high ratio of green fluorescence was emitted, suggesting a good cell–bioscaffold interaction as the presence of live cells (>90%) exceeded the ratio of dead cells. Quantitively the percentage of live cells was the highest in QC0.5 (98.57 ± 1.36%) hydrogel, followed by QC0.1 with 98.00 ± 2.14%, QC0.3 94.83 ± 4.95%, and finally, in GCL (94.16 ± 8.15%) and GECL, respectively. 

**Figure 8:**
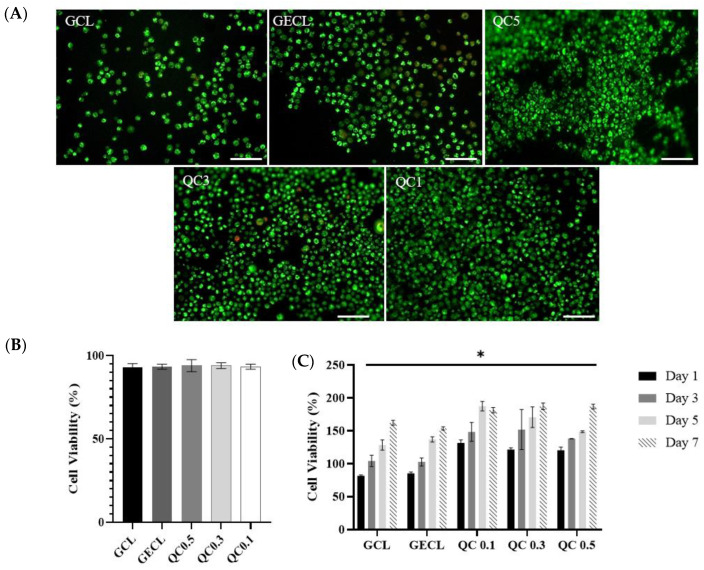
(**A**) The cellular compatibility of the scaffolds with human dermal fibroblasts prior to 24 h incubation at 37 °C. (**B**) The cell viability quantification of human dermal fibroblasts using LIVE/DEAD assay. (**C**) The quantification of cell proliferation from day 1 to day 7. * significant difference (*p* < 0.05).

Whereas in the MTT cell proliferation assay, there is a consistent increase in cell viability for all the Gelastin hydrogels from day 1 to day 7, there is no significant difference between the groups; however, it can be seen that the QC-embedded group conveys high cell viability toward day 7 (Figure 8B). The high cell viability observed in all the hydrogels indicates that the material is non-cytotoxic and can support cell growth for an extended period of time (7 days).

## 3. Conclusions

The incorporation of genipin is one of the most vital steps in the optimization of this novel hydrogel for the preparation of elastic and resistant gels as gelatin has low mechanical properties, whereas genipin is a stable, highly biocompatible crosslinker which aids with gelatin’s lack of mechanical strength. The findings of this study revealed that the hybrid gelatin–elastin injectable hydrogel holds great potential to be used in future provisional biotemplate applications as it has great physicochemical properties and biocompatibility. The amorphous and soft gel-like properties of the hydrogels obtained from the XRD and rheology studies made it possible for the hydrogels to be implanted easily onto the wound regardless of the complexity of the shape of the defect area. Whereas the WVTR obtained are theoretically able to maintain good moisture retention without excessive dehydration of the wound, and the swelling ratios are potentially sufficient for the hydrogels to absorb wound exudates, maintaining a suitable microenvironment for wound healing purposes. Furthermore, the injectable hydrogels are highly biocompatible with HDFs and are able to support cell proliferation in an extended time period of 7 days. Nevertheless, further studies will be carried out to determine the hydrogel’s potential antioxidant properties.

## 4. Materials and Methods

### 4.1. Materials

Gelatin supplied from Nitta-Gelatin Ltd. (Japan headquarter) is a high-grade quality, low-endotoxin unit essential for diminishing immune rejection post-implantation. It is currently manufactured at Nitta-Gelatin Ltd. (India branch) and is certified halal, originating from a buffalo’s raw bone. Quercetin (QC) was purchased from Sigma-Aldrich (St. Louis, MO, USA) and utilized without further purification. Elastin was procured from the Faculty of Science and Technology, Universiti Kebangsaan Malaysia (FST, UKM) and Genipin was purchased from FUJIFILM Wako, Osaka, Japan. Pharmaceutical-grade solvents and reagents were used in this study and were used as received.

The location for this study was the Centre for Tissue Engineering and Regenerative Medicine (CTERM), Faculty of Medicine, and several tests were run at FKAB, UKM, and iCRIM UKM. The study design was approved by the Universiti Kebangsaan Malaysia Research Ethics Committee (UKM PPI/111/8/JEP-2021-301).

### 4.2. Optimization of Gelastin Hydrogel

#### 4.2.1. Gelation Time

Prior to successful fabrication, during the optimization phase, gelatin was dissolved in distilled water (dH_2_O) at different time points of 0, 15, 30, and 60 min to obtain the most solubilized hydrogel solution. The hydrogels were pre-crosslinked using a 0.1% (*w/v*) concentration of genipin. As performed by Cao et al., the polymerization time for each formulation was determined via inverted tube test analysis at room temperature (27 °C ± 10 °C) [32]. An image of the gross appearance was taken using a digital camera (Nikon, Tokyo, Japan).

#### 4.2.2. Dose–Response (Cell Toxicity)

An MTT assay (3-(4,5-Dimethylthiazol-2-yl)-2,5-diphenyltetrazolium Bromide Tetrazolium) was utilized. This assay measures the reduction of yellow MTT (3-(4,5-dimethylthiazol-2-yl)-2,5-diphenyltetrazolium bromide) to an insoluble blue formazan product using mitochondrial succinate dehydrogenase. Non-viable cells cannot convert MTT into “purple” formazan; hence, it is considered a colorimetric assay that presumably serves as a marker for cell viability [33]. This study was performed in compliance with ISO 10993-5:2009, the in vitro safety study. Briefly, cells were seeded into 96-well plate at a seeding density of 5 × 103 cells/well and incubated at 37 °C for 24 h or before 80% confluency was reached. The quercetin was tested in triplicate at concentrations of 0.0625, 0.125, 0.25, and 0.5 mg/mL in a complete growth medium (FDC). The complete growth medium was then replaced with 200 μL/well of the test material (quercetin solution) in a 96-well plate containing healthy culture and incubated for another 24 h at 37 °C in a CO_2_ incubator. After 24 h incubation, the quercetin solution, washed twice with PBS, was discarded and replaced with 200 ul DMEM supplemented with 20 μl 5 mg/mL MTT solution and incubated (4 h at 37 °C) in a CO_2_ incubator. The purple formazan crystals were solubilized in dimethyl sulfoxide (DMSO), and the optical density was determined at 570 nm. The cell viability percentage was calculated as the given equation:

$$Cell\;Viabilty\;\left(\%\right)=\frac{\mathrm{ODt}-\mathrm{ODb}}{\mathrm{ODnc}}\times 100$$

where ODt: OD of the test substance, ODb: OD blank sample, and ODnc: OD of the negative control

#### 4.2.3. Preparation of Gelastin (Gelatin–Elastin) Hydrogel

**Scheme 1:**
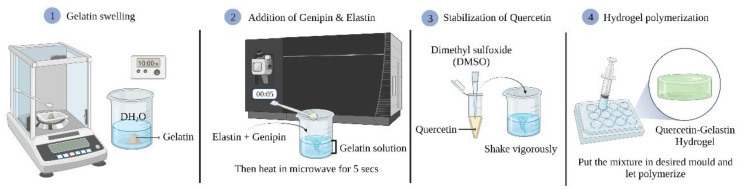
Schematic representation of the quercetin-embedded Gelastin hydrogel fabrication process.

Briefly, gelatin powder was llowed to swell in distilled water (dH_2_O) (room temp. 27 °C ± 10 °C, 10 min). Then, 0.2% (*w/v*) elastin was added to the gelatin solution, followed by 0.1% (*w/v*) genipin as the crosslinking agent. The mixture was then heated in a microwave (Samsung MI600N, 230 V, 50 HZ, 600 watt) for 5 s (47 °C ± 10 °C) and shaken vigorously to obtain a homogenous mixture. To stabilize the quercetin, the powder was solubilized in 50 ul dimethyl sulfoxide (DMSO) per 10 mL (0.5:10 000 ratio) hydrogel fabrication, and added dropwise in the heated Gelastin solution while shaking vigorously. The mixture was resuspended using a Pasteur pipette and transfer into desired mold and allow it to polymerize. Scheme 1 demonstrates a schematic illustration of the fabrication process. The non-crosslinked, crosslinked, and quercetin-embedded hydrogels were labeled GNC, GCL, GENC, GECL, QC 0.1, QC 0.3, and QC 0.5, respectively.

### 4.3. Physico-Chemical Characterization of Gelastin Hydrogels

#### 4.3.1. Energy-Dispersive X-ray

The elemental contents on the surface of the hydrogel were analyzed via Energy-Dispersive X-ray (EDX) (Phenom, Eindhoven, Netherlands) microanalysis. The commercially available gelatin acted as the control.

#### 4.3.2. Fourier Transform Infrared Spectrophotometry

Fourier transform infrared (FTIR) spectroscopy was utilized to characterize the hydrogels (PerkinElmer, Waltham, MA, USA). The FTIR spectra were obtained from a portion of gelatin flakes, quercetin, genipin, and elastin powder and tested on the FTIR spectrophotometer. Measurements were performed at 4000–500 cm^−1^ at a resolution of 2 cm^−1^ per point at room temperature.

#### 4.3.3. X-ray Diffraction Study

The X-ray Diffraction (XRD) characterization of the sample was performed using radiation at room temperature in the –2 scan mode using advanced X-ray diffractometer equipment (Bruker AXS GmbH, Karlsruhe, Germany). The diffraction patterns were recorded via XRD analysis using CuKα radiation (λ = 1.542 Å) at 35 kV and 10 mA. The sample was scanned with 2θ (where θ is the Bragg angle) varying from 10° to 70° in a continuous mode. The result obtained were analyzed using integrated software to identify the specific peaks. 

#### 4.3.4. Microporous Structure Study

Scanning Electron Microscopy (SEM), operated at 15 kV, was utilized to observe the sample’s surface topography and cross-section microstructure. The pore size of the sample was measured randomly using measurement software. Field emission SEM was used to observe the fibrous structure under higher magnification, whereas the solvent replacement method, as previously optimized by Mun et al. [34], was used to evaluate the hydrogel porosity. The initial weight (M_1_) of the lyophilized hydrogels was recorded prior to the 99.5% EtOH immersion for 24 h. Then, the excess ethanol was slowly blotted using filter paper (Whatman^®^, No. 42, Merck, Darmstadt, Germany), and the hydrogel (M2) weight was noted. The percentage of porosity was calculated using the following formula:

$$Porosity\;\left(\%\right)=\frac{M2-M1}{\rho V}\times 100\;$$

where ρ: density of 99.5% EtOH and *V*: volume the of hydrogel.

#### 4.3.5. Contact Angle

The wettability of the sample was determined using dH_2_O, which was compared to the control (without crosslink). Briefly, 10 microliters of dH_2_O were dropped onto the surface of the hydrogel and the angle was analyzed using the ImageJ application (NIH, Bethesda, MD, USA).

#### 4.3.6. Water Vapor Transmission Rate (WVTR)

This method was adapted from Rui et al., 2016 and validated based on the American Society for Testing and Materials (ASTM) standard [25,26]. Briefly, the hydrogels were placed on the opening of a glass vial that contained 10 mL of dH_2_O. The samples were to be placed in a controlled environment (5% CO_2_ at 37 °C). The water vapor transmission rate was recorded and calculated as shown below:

$$WVTR\;\left(g/m^{2}\cdot \mathrm{hour}\right)=\frac{\left(W_{i}-W_{f}\right)}{\left(A\times time\right)}$$

where *W_i_*: the initial weight, *W_f_*: the final weight, and *A*: the surface area of the glass vial.

#### 4.3.7. Degree of Crosslinking

The crosslinking degree of the samples was determined via a ninhydrin assay, and non-crosslinked hydrogels were used as a control. Briefly, 0.1 mg glycine was weighed and diluted in dH_2_O to obtain the serial dilution for the glycine standard (0.006, 0.0125, 0.025, 0.05, and 0.1 mg/mL). Then, 10 mg of the individual test sample was placed in an Eppendorf tube with 1 mL of ninhydrin reagent (Sigma-Aldrich, St. Louis, MO, USA), in a dark environment. The tubes were vortexed, and then, boiled (at 100 °C for 2 min), followed by a cooling step. One milliliter of 95% EtOH was added to the samples and glycine standards (Sigma-Aldrich, St. Louis, MO, USA). It was transferred into a 96-well plate and the absorbance was read at 570 nm on the spectrophotometer. The formazan formed was purple with the higher amine group and slightly yellow with the lower amine group.

#### 4.3.8. In Vitro Biodegradation 

The samples were standardized to a 25 mg initial weight, placed in a culture plate, and immersed in 0.0006 mg/mL of collagenase type I in DPBS. The biodegradation was evaluated by weight loss in the solution at 37 °C at different time points. The percentage of weight loss was calculated as shown below:

$$Biodegradation\;rate\;\left(\mathrm{mg}/\mathrm{hour}\right)=\frac{\left(W_{i}-W_{f}\right)}{t}\;$$

where *W_i_*: weight initial, *W_f_*: weight final, and t: time.

#### 4.3.9. Swelling Ratio Analysis

The samples were placed on a culture plate and immersed in Phosphate-Buffered Saline (PBS) at 37 °C (1 h and 24 h). Before being immersed in the PBS, the sample had to be weighed in dry form (Wd). At different time points, the PBS was removed from the culture plate. Prior to that, the liquid residual was removed by blotting the samples on filter paper and weighing them to obtain the swollen weight (Ws). The *swelling ratio* (SR) was experimentally determined using the following formula:

$$Swelling\;Ratio\;\left(\%\right)=\frac{\left(W_{s}-W_{d}\right)}{W_{s}}\times 100$$

where *W_s_*: the swollen weight of the hydrogels and *W_d_*: the dry weight of the hydrogels

The swelling ratio is the fractional increase in the weight of the hydrogel caused by water absorption [35].

#### 4.3.10. Resilience

Briefly, pressure was applied to the hydrogels with a 300 g metal load for 2 min, and then, the hydrogels were immersed in Phosphate-Buffered Saline (PBS) and left for 2 min. Prior to that, the area of hydrogel was captured and tabulated, and the same steps were repeated for the hydrogels after 2 min of bloating in the PBS. The data were then analyzed using the ImageJ application (NIH, Bethesda, MD, USA). The percentage of resilience was determined using the following equation:

$$Resilience\;\left(\%\right)=\frac{\left(A_{r}-A_{o}\right)}{A_{o}}\times 100$$

where *A_r_*: the area after rehydration, and *A_o_*: the area before rehydration.

#### 4.3.11. Rheological Analysis

An AR2000 rheometer (TA Instruments) with a 20 mm parallel plate accommodates a temperature-controlled Peltier plate. The rheological characterization was conducted using a 1% strain, and a 0.1 rad/s to 100 rad/s angular frequency at a constant temperature of 25 °C. The storage G′ modulus and loss modulus G″ were obtained.

#### 4.3.12. Cell Isolation and Culture

Redundant skin samples were obtained from all consenting healthy patients undergoing abdominoplasties such as appendicitis, abdominoplasty, or facelift. In brief, skin samples (3 cm^2^) were cleaned of unwanted fragments such as fat, hair, and debris, and minced into small pieces (approximately 2 mm^2^). The skin was digested in 0.6% collagenase type I (Worthington, Lakewood, NJ, USA) for 5–6 h in a 37 °C incubator shaker, followed by cell dissociation using 0.05% Trypsin-EDTA (Gibco, Carlsbad, CA, USA) for 8–10 min. The human dermal fibroblasts were obtained and cultured in fibroblast growth medium (F-12: Dulbecco’s modified eagle medium (Gibco, Carlsbad, CA, USA) supplemented with 10% fetal bovine serum (FBS) (Gibco, Carlsbad, CA, USA).

#### 4.3.13. LIVE/DEAD and Cell Attachment Assay

A LIVE/DEAD™ Cell Viability Assay (Invitrogen, Waltham, MA, USA) was utilized to analyze the cytotoxic effect of the elastin–gelatin hydrogel, according to the manufacturer’s protocol. Briefly, HDFs were seeded on the hydrogels prior to one day before incubation with calcein and EthD-1 (ratio 1:4) in PBS for 30 min at 37 °C; then, they were gently washed with PBS afterward. The cells were visualized using a fluorescence microscope (CLSM; Nikon). The live and dead cells were stained in green and red, respectively. The cell attachment assay was determined using the Trypan blue dye exclusion method. Briefly, the HDFs were directly cultured on the hybrid hydrogels prior to the 24 h incubation period. The cultured media were obtained and centrifuged for 5 min at 5000 rpm at 37 °C (Hettich Zentrifugen, Föhrenstraße, Tuttlingen, Germany). The supernatant was discarded, and the pellet was resuspended in 2 mL of DPBS (Sigma-Aldrich, St. Louis, MO, USA). A total of 10 μL of the cell suspension diluted with 10 μL Trypan blue (Sigma-Aldrich, St. Louis, MO, USA hemocytometer (Optik Labor, 0.100 mm, Görlitz, Germany)) was used under a light microscope (Olympus CK40, Tokyo, Japan) to obtain a visualization of the unattached cells. The cell attachment percentage was determined using the given equation:

$$\mathrm{Cell}\;\mathrm{Attachment}\;\left(\%\right)=\frac{Ni-Nd}{Nd}\times 100$$

where *Ni*: initial cell seeding, whereas *Nd*: the number of cells in DPBS.

HDF viability was evaluated on days 1 and 7 using a 3-(4, 5-dimethylthiazol-2-yl) -2,5-diphenyltetrazolium bromide (MTT) assay kit according to the manufacturer’s recommendations. Briefly, the scaffolds containing cells were fed with fresh medium (100 μL) and MTT reagent (10 μL) and incubated for 4 hr at 37 °C. Then, 100 μL dissolution reagents were added, followed by incubation for 4 hr at 37 °C. Absorbance was measured at 565 nm.

#### 4.3.14. Statistical Analysis

The data are shown as the mean ± SD. The mean between groups was compared via a one-way ANOVA test using SPSS software. A *p*-value ≤ 0.05 was considered significantly different.

## Figures and Tables

**Table 1:** Physical characteristics of crosslinked Gelastin hydrogels made of different concentrations of gelatin and with different swelling times, *n =* 6.

Gelatin	5% (*w/v*)				7% (*w/v*)			
**Swelling Point**	Direct	15 min	30 min	60 min	Direct	15 min	30 min	60 min
**Appearance**								
**1. Homogeneity**	No	Yes	Yes	Yes	No	Yes	Yes	Yes
**2. Clarity**	No	Yes	Yes	Yes	No	Yes	Yes	Yes
**Polymerization** (within 3 min)	No	No	No	No	Yes	Yes	Yes	Yes
**Odor**	No	No	No	No	No	No	No	No

**Table 2:** The crystallinity and amorphous study of the hydrogels via X-ray Diffraction (XRD) analysis.

X-ray Diffraction (XRD)		
Hydrogel	Crystallinity	Amorphous
GNC	18.1%	81.9%
GCL	41.8%	58.2%
GENC	37.7%	62.3%
GECL	44.8%	55.2%
QC 0.5	46.7%	53.3%
QC 0.3	42.0%	58.0%
QC 0.1	40.3%	59.7%

**Table 3:** Elements analysis (percentage of carbon, hydrogen, and nitrogen) of hybrid hydrogels via Energy-Dispersive X-ray (EDX).

Energy-Dispersive X-ray (EDX)			
Hydrogel	C (%)	O (%)	*n* (%)
GNC	58.2 ± 0.66	25.2 ± 2.01	16.6 ± 2.18
GCL	58.7 ± 2.50	25.2 ± 2.38	16.6 ± 2.01
GENC	58.5 ± 0.50	21.6 ± 2.08	17.8 ± 1.52
GECL	60.0 ± 2.12	25.1 ± 3.70	16.0 ± 2.34
QC 0.5	62.9 ± 2.50	18.7 ± 1.96	18.3 ± 1.79
QC 0.3	62.6 ± 4.81	19.2 ± 2.38	18.2 ± 2.56
QC 0.1	60.2 ± 2.12	22.9 ± 3.70	18.0 ± 2.34

**Table 4:** Resilience data of gelastin-hydrogels.

Resilience Data			
Hydrogel	Initial (cm^2^/ mg)	After Compression (cm^2^/ mg)	After PBS Treatment (cm^2^/ mg)
GNC	1.042	0.978	1.045
GCL	0.984	0.825	0.898
GENC	1.03	0.876	1.117
GECL	0.955	0.895	0.943
QC 0.5	0.957	0.814	0.866
QC 0.3	0.963	0.848	0.961
QC 0.1	0.932	0.839	0.904

## Data Availability

Not applicable.
